# Pleiotropic Effects of Metformin on the Antitumor Efficiency of Immune Checkpoint Inhibitors

**DOI:** 10.3389/fimmu.2020.586760

**Published:** 2021-02-02

**Authors:** Wenhui Liu, Ying Wang, Jianquan Luo, Mouze Liu, Zhiying Luo

**Affiliations:** ^1^ Department of Pharmacy, The Second Xiangya Hospital, Central South University, Changsha, China; ^2^ Institute of Clinical Pharmacy, Central South University, Changsha, China

**Keywords:** metformin, immune check inhibitors, PD-1, PD-L1, pleiotropic, gut microbiome

## Abstract

Cancer is an important threat to public health because of its high morbidity and mortality. In recent decades, immune checkpoint inhibitors (ICIs) have ushered a new therapeutic era in clinical oncology. The rapid development of immune checkpoint therapy is due to its inspiring clinical efficacy in a group of cancer types. Metformin, an effective agent for the management of type 2 diabetes mellitus (T2DM), has shown beneficial effects on cancer prevention and cancer treatment. Emerging studies have suggested that metformin in combination with ICI treatment could improve the anticancer effects of ICIs. Hence, we conducted a review to summarize the effects of metformin on ICI therapy. We also review the pleiotropic mechanisms of metformin combined with ICIs in cancer therapy, including its direct and indirect effects on the host immune system.

## Introduction

Over the past decade, cancer has emerged as one of the most important threats to public health because of its high morbidity and mortality (1). Although there is a long way to go before cancer is completely conquered, the pace of scientists’ fight has never slowed down. The current therapeutic strategies for cancer have made a great breakthrough compared with those several decades ago. ICIs such as the inhibitors of cytotoxic T-lymphocyte antigen-4 (CTLA-4; ipilimumab), programmed cell death receptor-1 (PD-1; nivolumab, pembrolizumab), and its ligand (PD-L1; atezolimumab, avelumab, and durvalumab) have revolutionized cancer treatment (2). Their mechanism involves blocking inhibitory receptors and then reactivating cytotoxic T cells to kill or destroy cancer cells, thus resulting in long-lasting tumor responses (3). ICIs exhibit unparalleled therapeutic efficacy in multiple cancer types and are rapidly transforming oncology practices.

Metformin is one of the most widely used drugs for patients with T2DM and has several obvious advantages such as established treatment efficacy, a good safety profile, and low cost. Recent evidence indicates novel pleiotropic actions of metformin. In addition to its consolidated role in T2DM management, it displays antifibrotic, antioxidant, antiaging, and cardio- and nephron-protective effects (4). Accumulating evidence suggests a preventive role of metformin in multiple cancer types including pancreatic, colorectal, prostate, and hepatocellular carcinoma; metformin intake results in decreased cancer incidence and mortality (5). The anticancer activity of metformin has also been widely studied under both *in vivo* and *in vitro* conditions. This activity is mediated particularly through the direct inhibition of the AMPK/mTOR pathway and indirectly influences its glucose-lowering properties and anti-inflammatory effects (6).

Although great success has been achieved in clinical oncology therapy, the current ICIs face two challenging problems: a low response rate and a higher rate of occurrence of immune-related adverse effects (irAEs) (7, 8). Metformin could convert immunotherapy resistance in patients who received metformin plus anti-PD-1 treatment by preventing the presentation of PD-1+/CD8+ T cell infiltrates after drug withdrawal (9). Hence, the clinical benefit of metformin along with ICIs in oncology therapy needs to be evaluated to improve the response rate of ICIs. A previous study found that metformin combined with anti-PD-1 therapy had a potential benefit in mouse models, as the metformin-induced reduction of the tumor hypoxia enhanced the efficacy of PD-1 blockade (10). Recently, more attention has been given to the T cell-mediated antitumor response of metformin in the immunosuppressive process of cancer therapy (1, 11). Extensive research has suggested that metformin has the potential to enhance antitumor immune responses in different types of cancers. Hence, we conducted this review to summarize the clinical benefits of metformin combined with ICIs in oncology therapy and reviewed the related pleiotropic mechanisms.

## Clinical Status and Predictive Biomarkers of ICI Therapy

ICIs represent a breakthrough in the treatment of advanced cancers. Unfortunately, factors including uncertain clinical efficacy, low overall effective rate (almost 20%), drug resistance, serious irAEs, and lack of biomarkers restricted the clinical value. What’s more, ICI therapy is costly. For example, each patient treated with ipilimumab incurs an annual cost of 120,000 Euros. Although ICI combined with chemotherapy can improve the response rate (40%–60%), this therapeutic strategy is associated with increased treatment costs and severe or potentially life-threatening irAEs (12). Such adverse reactions were observed in up to 80% of the patients who received the combination therapy (8). It is widely evidenced that ICI therapies have demonstrated a clinical response only in a fraction of patients with advanced cancer. Therefore, it is important to identify predictive and prognostic biomarkers to select patients who are expected to clinically benefit from these therapies.

Several prognostic biomarkers of ICI therapy have been previously recognized to predict interindividual differences in ICI treatment. The expression level of PD-L1 on tumor tissue was first shown to be the most likely predictive biomarker of PD1/PD-L1 therapy in various cancer types. Currently, only patients with PD-L1 TPS ≥ 50% can receive single-agent immunotherapy (pembrolizumab) as the first-line treatment in clinical practice. Such patients account for a maximum of 30% of all patients with advanced non-small cell lung cancer (NSCLC) (13). Although patients with higher PD-L1 expression levels show a higher likelihood of responding to ICIs, approximately 10% of patients with negative PD-L1 expression respond to PD1/PD-L1 therapy and some strongly-positive PD-L1 patients do not respond (14, 15). A recently published meta-analysis assessed the diagnostic accuracy of PD-L1 immunohistochemistry, tumor mutational burden (TMB), gene expression profiling (GEP), and multiplex immunohistochemistry/immunofluorescence (mIHC/IF) in predicting response to anti-PD-1/PD-L1 therapy, and mIHC/IF demonstrated higher positive predictive values (0.63) and positive likelihood ratios (2.86) than the other approaches (16).

Numerous studies have shown that factors other than those related to tumor tissue (e.g., tumor mutational burden, mismatch repair deficiency, and neoantigens), such as peripheral blood (e.g., lymphocytes and neutrophils) and other sites (gut microbiome) might affect the response to ICIs; such factors have been suggested as predictive biomarkers in previous studies (17). Pembrolizumab was approved by the FDA as the very first tissue-agnostic drug in patients with advanced solid cancer and conditions such as positive microsatellite instability-high (MSI-H) or DNA mismatch repair dificiency (dMMR) regardless of the tumor site or histology. However, the predictive abilities of these biomarkers have not yet been verified adequately by prospective and randomized clinical trials. Moreover, it would be difficult to predict responses using a single biomarker because of the complexity of the tumor immune system and autoimmunity.

## Antitumor Efficiency of Metformin

In 2005, data from a retrospective study with a large sample size showed that intake of metformin was associated with a reduced risk (23%) of cancer in patients with T2DM (18). Sakoda et al. found that patients with T2DM had a 43% lower risk of lung cancer and 52% lower risk of lung cancer if they took metformin for more than five years (19). Since then, an increasing number of researchers have focused on the antitumor effects of metformin. In recent years, there has been much evidence that metformin could be used to prevent or slow the growth of certain cancers and that individuals taking metformin have a reduced risk of developing certain cancers and dying from them (5, 20).

Several clinical trials have been conducted to evaluate the effects of neoadjuvant metformin on several cancer types. An increase in apoptosis and a decrease in Ki67 scores (a biomarker of tumor proliferation) were observed in patients with breast cancer who used metformin in the neoadjuvant setting (21). Data from other prospective clinical trials also showed a reduction in the calculated Ki67 index in patients with neoadjuvant intervention with metformin in prostate cancer (22) and endometrial cancer (23). However, two presurgical trials found no reduction in the Ki-67 scores in patients with breast cancer who took metformin before surgery (24, 25). Although a large part of the above results supports the benefit of neoadjuvant metformin, more large-scale, randomized, and controlled clinical trials are required to further validate the clinical value of preoperative metformin in different cancer types.

Recent meta-analyses have shown that metformin acts as a useful adjuvant in cancer chemotherapy, particularly in patients with colorectal, prostate, and pancreatic cancer (26, 27). More encouraging evidence was obtained by Marsha et al. who found that metformin in combination with panhematin suppressed the tumor growth of triple-negative breast cancer (TNBC) (28). Panhematin is an inhibitor of the heme-binding transcription factor (BACH1) that displays increased expression in the tumors of patients with TNBC. However, metformin may have pleiotropic functions in different cancer types. The addition of metformin dose not improve the outcomes of patients with metastatic pancreatic cancer [treated with standard systemic therapy] (29) or those of patients with advanced pancreatic cancer [treated with gemcitabine and erlotinib] (30). Despite limitations, the combined data from laboratory and observational studies support the use of metformin as an adjuvant for cancer treatment in cancers with the strongest evidence base. All these studies have provided sufficient theoretical evidence for the antitumor effects of metformin. The specific clinical applicability of metformin in cancer therapy remains to be verified by extensive clinical trials.

## Metformin Influences the Antitumor Efficiency of ICIs

Although metformin monotherapy had little therapeutic benefit in highly aggressive tumors, the combination of metformin and anti-PD-1/PD-L1 blockade resulted in improved intratumoral T-cell function and tumor clearance *in vivo* (10). Based on the promoting effects of metformin on anti-PD-1/PD-L1 therapy in preclinical studies, more scientists are concentrating on the antitumor effects of metformin in combination with ICIs (as summarized in **Table 1**). Several studies observed favorable treatment outcomes (objective response rate, disease control rate, median progression-free survival, and median overall survival) in patients who received metformin in combination with ICIs without reaching a statistically significant trend (1, 11, 37). The subgroup analysis found a statistically significant association between metformin use and OS in obese patients with a BMI >25 kg/m^2^; the strength of the association was higher in patients with a BMI >30 kg/m^2^ (31). However, one study found worse treatment outcomes in patients who took metformin plus ICIs with a nonsignificant trend (32). Furthermore, another recent study showed that patients with lung cancer concomitant with diabetes mellitus had a higher risk of inflammatory bowel disease during the combined therapy of nivolumab plus metformin (35).

**Table 1:** Clinical trials using metformin combined immune checkpoint inhibitors (ICIs) for treatment of cancers.

Tumor site	Sample size	Outcomes	Ref.
metastatic melanoma	55 patients, 22 (40%) patients used metformin combined ICIs	ORR (68.2% vs. 54.5%, P = 0.31); DCR (77.3% vs. 60.6%, P = 0.19). Median OS (46.7 M vs. 28 M); Median PFS (19.8 M vs. 5 M); New metastatic sites (0.59 vs. 1.51, p=0.009)	(31)
NSCLC	50 patients, 21 (42%) patients used metformin combined ICIs	ORR (41.1 vs 30.7%, p = 0.4); DCR (70.5 vs 61.6%, p = 0.5); Median OS (11.5 vs 7.6 months, p = 0.5); Median PFS (4.0 vs3.0 months, p = 0.6)	(32)
NSCLC	434 patients, 74 (17%) patients used metformin combined ICIs	A tendency to better OS in metformin users only in patients with a BMI >25 kg/m2 and the strength of the association was higher in patients with BMI >30 kg/m^2^	(33)
NSCLC	224 patients, *18 (8*%) patients used metformin combined ICIs	Median PFS (3.3 vs. 6.0, P=0.562); Median OS (10.6 vs.13.1 , P= 0.440)	(34)
metastatic cancer	210 patients, 23 (11%) patients used metformin combined ICIs	Clinical benefit rate (17.3% vs. 28%, P= 0.28)	(35)
Advanced Melanoma	330 patients, 34 (10.3%) patients used metformin combined ICIs	Median PFS (11.1 vs. 5.6 months; P= 0.36) Median OS (27.6 vs. 26.0 month; P= 0.48)	(36)

ORR, objective response rate; DCR, disease control rate; OR, overall survival; PFS, progression free survival.

These studies raise the question regarding the effect of metformin in significantly enhancing the antitumor effects of ICIs in xenograft models and not in clinical studies. We speculate that it is effective but only in a specific category of patients, such as obese patients. Ongoing clinical trials continue to examine the antitumor effects of metformin along with ICIs (as shown in **Table 2**) and refine our understanding of its mechanisms of action. Hence, large-scale prospective clinical trials and real-world studies are required to investigate the synergistic effect of metformin and ICIs before they can be recommended as routine additive therapy and to identify patients who would benefit the most from combination therapy.

**Table 2:** Ongoing clinical trials of metformin combined immune checkpoint inhibitors (ICIs).

Trial number	Phase	ICIs	Disease	Primary Purpose	Sponsor/Investigator
NCT03800602	Phase 2	Nivolumab	Refractory MSS Metastatic Colorectal Cancer	Treatment	Emory University
NCT03048500	Phase 2	Nivolumab	Stage III-IV NSCLC	Treatment	Northwestern University
NCT03311308	Phase 1	Pembrolizumab	Advanced Melanoma	Treatment	Yana Najjar
NCT04414540	Phase 2	Pembrolizumab	Head and Neck Squamous Cell Carcinoma	Treatment	Trisha Wise-Draper
NCT03618654	Phase 1	Durvalumab	Squamous Cell Carcinoma of the Head and Neck	Treatment	Sidney Kimmel Cancer Center at Thomas Jefferson University

## Possible Mechanisms of Metformin in Enhancing the Antitumor Effects of ICI Treatment

This section contains four subsections that review the pleiotropic effects of metformin on the antitumor action of ICIs.

### Metformin Influences ICI Efficiency by Adjusting the Intestinal Microbial Communities and Microbial Metabolites

The gut microbiome plays a variety of extremely important roles in host function, including innate and acquired immune responses, both locally and systemically (33). Accumulating evidence implies that the human gut microbiota produces dozens of metabolites, which can have crucial and systemic effects on the host (34). These metabolites are increasingly recognized as an essential part of human physiology and have profound effects on immune function and dysfunction (38). Short-chain fatty acids (SCFAs), the abundant metabolites produced by intestinal microbiota, have been demonstrated to be important drivers in the induction and activity of regulatory T cells (Tregs) (36). These metabolites activate the immune system by binding to the aryl hydrocarbon receptor (AHR); enhance the intestinal epithelial barrier; stimulate gastrointestinal motility; trigger the secretion of gut hormones; exert anti-inflammatory; antioxidative, or toxic effects in the systemic circulation; and putatively modulate the gut microbial composition (39).

The gut microbiome is widely recognized as being closely related to the occurrence and development of a variety of cancer types, such as gastric and colorectal cancer in the epithelial barrier and sterile tissues. Moreover, it could significantly influence the response and toxicity of various forms of cancer therapy (40). The antitumor effects of ICIs can be manipulated by altering the microbiota composition. Using murine models, scientists initially demonstrated that the antitumor effects of ICIs depend on the gut microbial communities (41). Thereafter, mounting data from human cohorts have further verified the key role of the gut microbiome in regulating the response to ICI immunotherapy. Among the patients who responded to ICI therapy, the diversity of the gut microbiome was significantly increased in those patients who had more abundant *Akkermansia muciniphila* (*A. muciniphila*), *Bifidobacterium* spp., *Alistipes*, *Bifidobacterium longum*, *Collinsella aerofaciens*, and *Enterococcus faecium* (42, 43). Administration of the gut microbiome cocktail was sufficient to increase the anti-PD-L1 efficacy significantly in the nonresponder group of mouse models.

The use of antibiotics might destroy the gut microbiome and impair the antitumor efficiency of ICIs by damaging the delicate balance of bacteria in the gut (44). A large number of prospective and retrospective studies have been conducted to investigate the influence of antibiotic use on ICI therapy. These studies found a lower efficacy of immunotherapy when antibiotics were coadministered with or before ICI therapy (43, 45–47). Overall, the influence of the gut microbiome on the therapeutic efficacy of ICIs is undeniable. Recent evidence has identified inosine as a key bacterial-derived metabolite acting through T cell-specific A2AR signaling to promote Th1 cell activation in a context-dependent manner (48). Hence, modifying the microbiota with the defined microbial consortia may provide a promising adjuvant therapy to ICIs in cancers.

Wu et al. first investigated the effect of metformin on the composition of the human gut microbiota and found that metformin treatment for two months significantly promoted the growth of a large part of the intestinal bacteria, especially the relative abundance of *γ-Proteobacteri*a and *Firmicutes* (49). Furthermore, a targeted analysis showed a significantly increased abundance of *A. muciniphila* in individuals who received metformin for four months (50). There is convincing evidence that metformin exposure induces a significant increase in the abundance of *A. muciniphila* and *Bifidobacterium* spp. under *in vitro* conditions in mouse model and in humans (51, 52). Therefore, we can speculate that metformin enhances the antitumor effects of ICIs by enhancing the community of the gut microbiome, especially the abundance of *A. muciniphila* and *Bifidobacterium* spp.

The gut microbiome is capable of influencing the antitumor effects of ICIs that are administered intravenously. Currently, the mechanism by which metformin influences the gut microbiota remains ambiguous. Metformin has been shown to restore the proportion of intestinal flora in a healthy way by providing an advantageous living environment for beneficial intestinal bacteria; thus, it plays a positive role in regulating the immune system. This is because the hypoglycemic effect of metformin occurs partly through a *B. fragilis*–*GUDCA*–intestinal *FXR* axis that improves metabolic dysfunction (53). Microbiota-generated metabolites and their cellular and molecular components are increasingly recognized as an essential part of human physiology with profound effects on immune function and dysfunction. Hence, we suspect that the gut microbiome or metformin affects the antitumor effects of ICIs through microbial metabolites (such as SCFAs and bile acids).

Metformin is usually taken orally and its oral bioavailability is between 50% and 60% (54). It is noteworthy that the metformin concentration in the gut lumen is 30–300 times greater than that in the serum. This implies that the mechanism of action requires higher concentrations so that enhancing the ICI effects may become relevant to the effects on the gut microbiota. Therefore, the degree to which the intestinal flora is altered by metformin is affected by host factors that are associated with the gut lumen concentration of metformin. This may account for the inconsistent clinical outcomes of metformin-combined ICI therapy.

### Metformin Directly Regulates Antitumor Immunity

**Figure 1:**
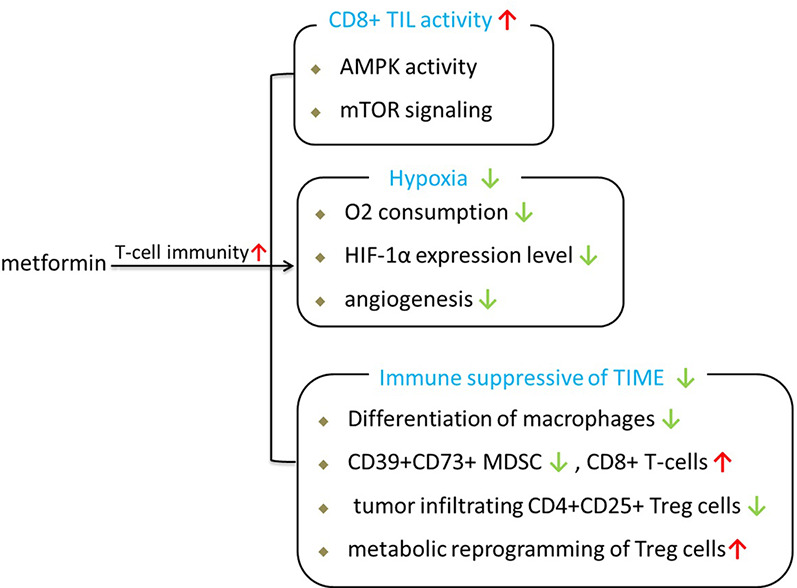
Beneficial effects of metformin on anti-tumor immunity.

Since T cells are the main effector of ICIs, T cell activity has been the ultimate goal of most FDA-approved tumor immunotherapies. Based on published researches, we consider that metformin can enhance antitumor immunity of ICIs through several approaches, as shown in **Figure 1**.

First, metformin is able to shift the suppressive state of T cells in the tumor. In vivo research demonstrated that metformin has much better antitumor effects in immune-competent mouse models than in immunodeficient models under the same conditions (55), implying that the effect of metformin is primarily mediated by antitumor immunity in clinical conditions. Hence, the antitumor effect of metformin is closely related to the immune response (56). A preclinical study showed that the anticancer effect of CD8+ T tumor-infiltrating lymphocytes (CD8TILs) is suppressed by the interaction between ICIs such as PD-1 and CTLA-4 expressed on CD8TILs and their ligands expressed on cancer cells; this process is referred to as immune exhaustion. CD8TILs are the target of metformin, which can counter this suppressed state and block immune exhaustion within tumor tissues *via* AMPK-mTOR signaling (57). Thus, the activity of CD8TILs in eliminating cancer cells in tumor tissues is enhanced (55).

Second, metformin can improve T-cell immunity by alleviating the intratumoral hypoxic state of the tumor microenvironment. Hypoxia-inducible factor (HIF-1), which accumulates under hypoxia, is the major transcription factor that activates genes involved in glucose absorption, glycolysis, and angiogenesis in tumors. It is now becoming clear that the hypoxic nature of the tumor microenvironment is associated with immunotherapy resistance because the T cells in the tumor microenvironment are at a metabolic disadvantage and repress oxidative metabolism (10). Metformin treatment can inhibit the oxygen consumption of tumors and the consequent generation of hypoxia, thereby enhancing the antitumor effects of PD-L1 (10). Metformin ameliorates the tumor hypoxic state accompanied by a significant reduction in the expression of both HIF-1α and angiogenesis-associated factors (AAFs), which are pro-angiogenic factors (58). Consistent with these findings, metformin can enhance antitumor immunity similar to CTLA4 immunotherapy, which not only blocks the inhibitory signal from cancer cells but also stimulates intrinsic T cell activation (59). These findings imply that the antitumor effects of metformin may play a role in the immune response against tumor progression.

Third, metformin has immunomodulatory effects and can be used in cancer immunotherapy by regulating the state of the tumor immune microenvironment (TIME). Tumor tissues contain many types of immune cells, including dendritic cells (DCs), natural killer (NK) cells, and macrophages as well as T and B lymphocytes from the adaptive immune system (60). The TIME includes a large number of immune suppressor cells, including tumor-associated macrophages (TAMs), myeloid-derived suppressor cells (MDSCs), and regulatory T cells (Treg cells). Tumors will lead to a TIME in an immune *suppressive* state to promote tumor growth during the process of solid tumor growth (61). Patients with an active TIME were shown to have better clinical outcomes than patients with a suppressive TIME within ICI treatment (62, 63).

During the past 2 years, many basic studies have shown that metformin has certain effects on regulating the tumor TIME and can partially activate the immune system. Metformin has been shown to inhibit the M2-TAM-driven catabolism of tryptophan to kynurenine, which is a characteristic immunosuppressive metabolite of the TIME that impedes T cell activity and promotes the development of Treg cells (64, 65). Metformin can reduce the frequency of circulating CD39+CD73+ MDSCs and increase the antitumor activities of circulating CD8+ T-cells *contemporaneously (* 66). Metformin generates sustained antitumor immunity by inhibiting the differentiation of naïve CD4+ T-cells to inducible Treg cells and attenuating tumor infiltrating CD4+CD25+ Treg cells by mTOR activation (67). Kunisada et al. further demostrated that metformin induced metabolic reprogramming toward glycolysis in Treg cells by decreasing the expression of Foxp3, mitochondrial-potential, and ROS production. Data from a recently published clinical trial further showed that low-dose metformin reprogrammed the TIME from an immune suppressive state to an equilibrated or activated state in patients with esophageal squamous cell carcinoma (68). As mentioned before, metformin exposure could create a more favorable tumor microenvironment for T cells and promote the activation of antitumor immunity.

### Metformin Exposure Decreases the Expression Levels of Immune Checkpoint Genes

Recently, a large number of studies have shown that the tumor PD-L1 expression level is a determinant and an important biomarker for the clinical response to anti-PD-1/PD-L1 therapy (69). The expression of PD-L1 in tumor cells affects T-cell immune responsiveness in a quantitative manner, and a high expression level of PD-L1 leads to an increased impairment of T-cell survival or activity (70). The expression of the PD-L1 gene is modulated by multiple factors and signals such as TP53, PTEN, and retinoblastoma protein (RP) in various types of tumors (71).

Increasing evidence has shown that metformin improves the antitumor effects of anti-PD-1/PD-L1 inhibitors by reducing the expression level of PD-L1. Cha et al. first reported that metformin promotes antitumor immunity by reducing PD-L1 expression *via* endoplasmic reticulum-mediated degradation in breast cancer (59). Another study further validated that metformin inhibited the expression of PD-L1 in an AMPK-dependent manner in endometrial carcinoma (56). A recently published study showed that metformin activated the Hippo signaling pathway to reduce the expression level of PD-L1 under *in vitro* and *in vivo* conditions in colorectal carcinoma; thus, it increased the efficacy of immunotherapy in colorectal carcinoma (72). The inhibition of PD-L1 expression, which negatively regulates immune function, enhances the cytotoxic activity of T cells, and increases antitumor immunity. These studies suggest that metformin may be used as an adjunct to enhance the antitumor effects of PD-1/PD-L1.

In contrast, according to a recently published study, metformin may increase the expression level of PD-L1 in liver kinase B1 (LKB1) wild-type NSCLC *via* AMPK-LKB1 signaling (71). Moreover, the results of a clinical study showed that metformin use was associated with significantly decreased expression of six (CTLA4, PDCD1, ICOS, BTLA, CD27, and LAG3) of the seven immune checkpoint genes in only those patients who had a high BMI; opposite results were found in the low BMI group (31). The expression levels of four (CTLA4, CD28, BTLA, and CD27) immune checkpoint genes were significantly increased in patients administered metformin. The discrepancy in the regulation of immune checkpoint genes may be relevant to the cancer cell types and the differences in the intrinsic aberrations of tumor suppressors and the tumor microenvironment.

### Direct Antitumor Effects of Metformin

**Figure 2:**
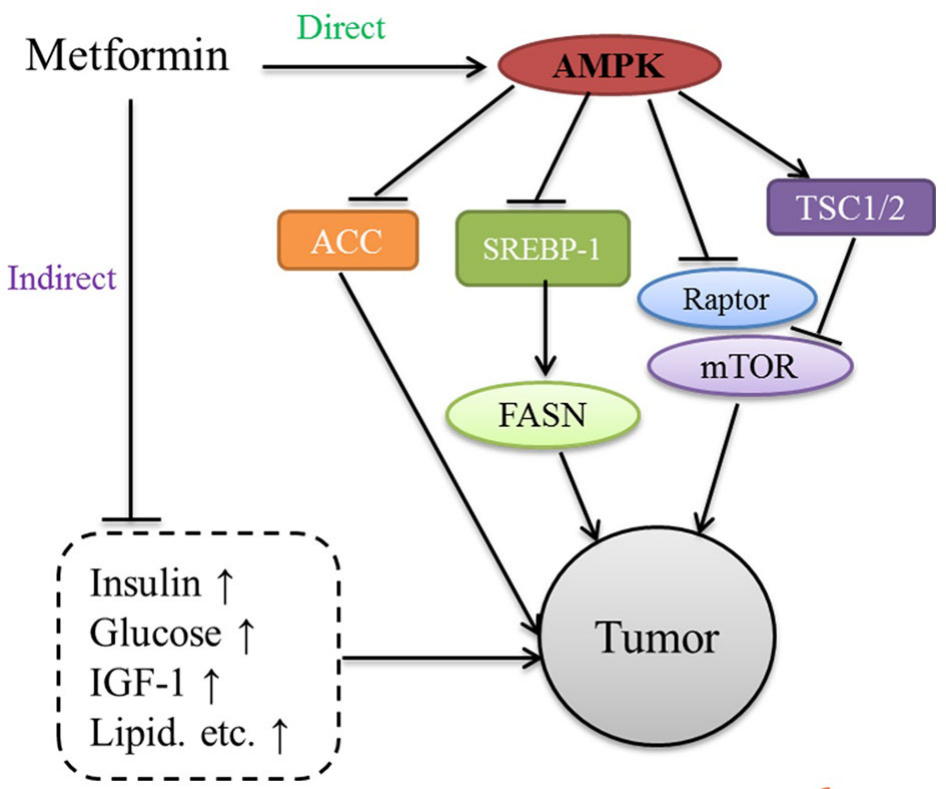
Processes of T-cell exhaustion and metabolic reprogramming.

We proposed in the previous sections that both epidemiological and clinical observation studies found that metformin could reduce the risk of cancer in patients with T2DM and improve the prognosis and survival rate of patients with cancer. Although scientists have investigated the antitumor mechanisms of metformin considerably, the mechanisms have not been fully elucidated. Over the past several years, these pleiotropic anticancer effects of metformin have been widely studied in numerous *in vitro* and *in vivo* studies at the molecular and cellular levels. The mechanisms of the antitumor effects of metformin can be classified as AMPK- and mTORC1-dependent or AMPK-, and mTORC1-independent, as shown in **Figure 2** (73).

Activation of AMPK inhibits mTOR signaling, the major regulator of cell growth and proliferation; this action of AMPK may be significantly involved in the anticancer mechanism of metformin’s action (74). The AMPK pathway may regulate the cell cycle by interacting with classical oncogenes and tumor suppressors such as c-MYC, NF-ĸB, and p53. Metformin has been shown to exert its antitumor effect *via* an AMPK-mTORC1-independent mechanism that has been attributed to decreased glucose and insulin levels and decreased production of biosynthetic precursors generated by the tricarboxylic acid cycle (73).

The anticancer effects of metformin enhance energy stress, inhibit epithelial-to-mesenchymal transition, and display antiproliferative and antiangiogenic effects, among others (75). Previous studies have shown that only a high metformin concentration (5-10 mM) could activate AMPK-dependent/independent signaling pathways under *in vitro* conditions. However, this concentration is higher than the plasma concentration of metformin (40 μM) in patients under physiological conditions (76). *In vitro* studies have shown that metformin at such low concentrations (micromolar range) is not sufficient to cause AMPK activation, although metabolic changes can be elicited. Therefore, the value of the results of these *in vitro* studies in clinical practice is limited.

### Metformin Regulates the Metabolic Preferences of Immune Cells

Under antigen stimulation, naïve T cells differentiate into effector T-cells or memory T cells *via* transcriptional regulation accompanied by metabolic reprogramming. Naïve T cells in a metabolically quiescent state use oxidative phosphorylation (OXPHOS) for energy production (77). These metabolic preferences were transformed to the phosphatidylinositol-3-kinase–protein kinase B–mammalian target of rapamycin (PI3K–AKT–mTOR) pathway during the T cell activation process (78). Effector T cells rely on aerobic glycolysis for their rapid expansion and effector functions.

**Figure 3:**
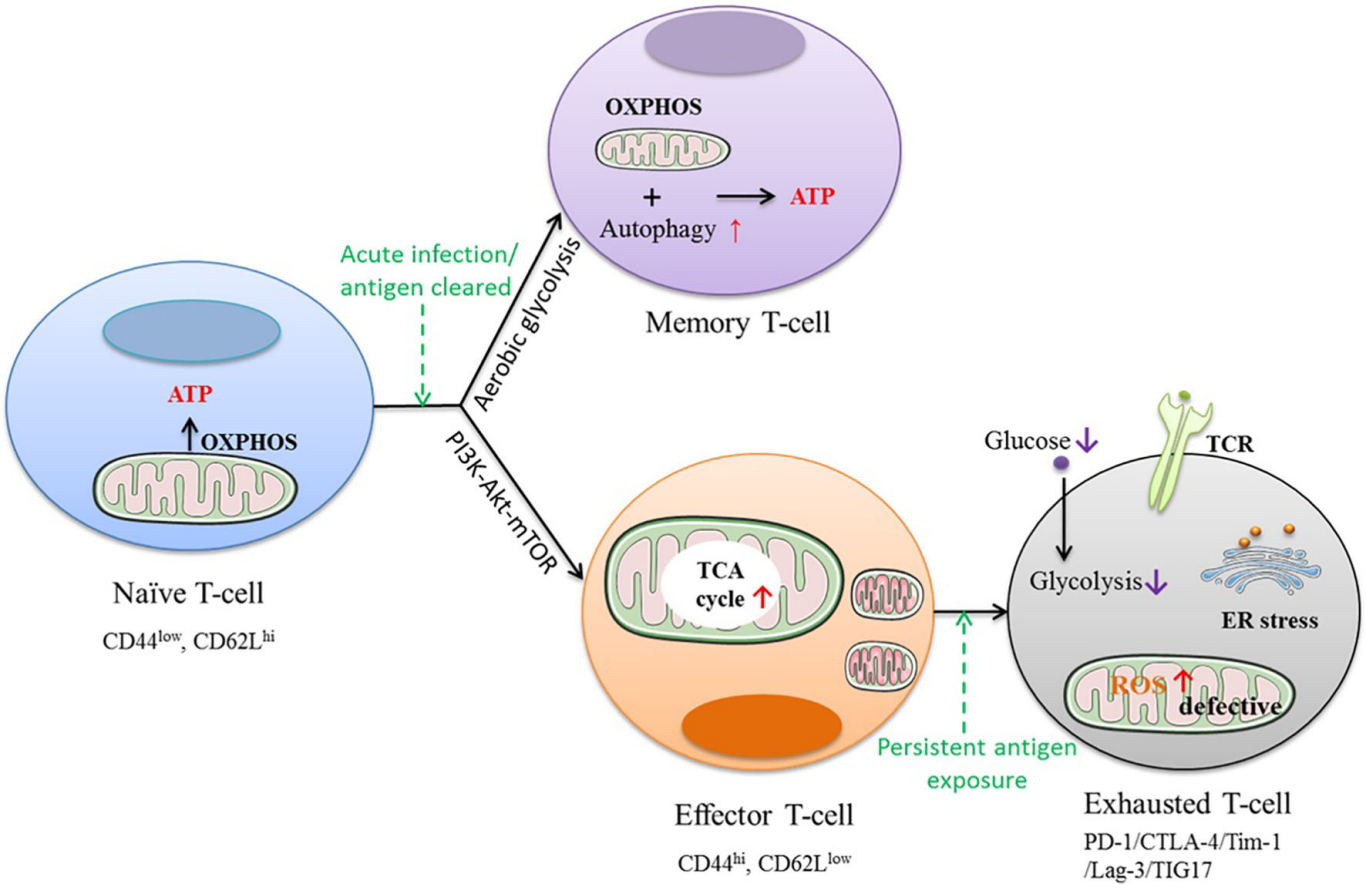
The direct and indirect anti-tumor effects of metformin.

During cancer development, CD8+ T-cells differentiate into a terminal differentiation state known as T-cell exhaustion because of chronic exposure to antigens and activation signals. T-cell exhaustion is characterized by elevated expression of inhibitory receptors such as PD-1, Tim-1, and Lag-3; activation of the endoplasmic reticulum (ER)-stress pathway; decreased development of effector cytokines; decreased effector function after T cell receptor (TCR) signaling stimulation and diminished proliferative capacity, as shown in **Figure 3** (79). Exhausted T cells have been shown to exhibit metabolic insufficiency with suppressed mitochondrial respiration and glycolysis (80). ICIs yield remarkable clinical outcomes by boosting the power of host immunity in cancer cell elimination. In contrast to effector T-cells, T-cell exhaustion poses hurdles to antitumor immunity during cancer treatment by ICIs (81). Hence, factors that affect the process of T-cell exhaustion could also influence the ICI response.

Metabolic remodeling of immune cells underlies improved antitumor immune responses and controls the antitumor immunity of ICIs. Critically, understanding the mechanism of metabolic reprogramming and the development of T-cell exhaustion has the potential to improve the clinical outcomes of current ICI therapies. Metformin could block T-cell exhaustion by increasing the number of CD8+ TILs and protect them from apoptosis and exhaustion, which was characterized by decreased production of IL-2, TNFα, and IFNγ (55). Metformin has been shown to act in a rapamycin-like manner to facilitate the shift of the metabolic state of T cells from a glucose-dependent anabolic state (effector T cells) to a catabolic state (memory T cells) by blocking mTOR signaling and restoring mitochondrial fatty acid oxidation (82). Metformin can reverse aberrant metabolism in an array of immune cell lineages and plays a critical role in regulating T cell subset differentiation by AMPK-mTOR-STAT signaling (83).

## Discussion

The clinical use of immunotherapy profoundly changed the paradigm of cancer treatment, and the dream of curing cancer is no longer out of reach. However, the long-term clinical benefits of current immunotherapy occur only in a small number of patients and many patients do not benefit (optimal outcomes have been limited to certain subsets of patients). This suggests that there must be other mechanisms limiting the immune response within the tumor. Hence, further preclinical and clinical studies are needed to confirm the potential effective combination therapy for cancer immunosuppression treatment.

Cancer cells frequently evade the antitumor response through various escape mechanisms such as immune checkpoint upregulation or tumor-promoting inflammation (78). Although ICIs overcome some mechanisms of tumor immune escape, many tumors achieve immune evasion and fail to respond (84). Along with pharmacodynamic studies of the concerned drugs, further molecular studies of the tumors would help elucidate any prognostic biomarkers showing response to these concurrent medications.

Although many anticancer studies and epidemiological studies have been conducted on metformin, they have always been confined to theoretical studies. When used in clinical studies, the results are usually unsatisfactory as is clinical transformation (20, 85). Therefore, metformin has not been used in the treatment of patients with cancer. The present evidence partly supports the use of metformin in combination with ICIs for improving the treatment of several cancers. This therapeutic schedule is also associated with a substantial risk of adverse effects (1, 11). Various approaches are underway to expand the benefits and improve the efficacy of these ICIs. Other drugs, including nonsteroidal anti-inflammatory drugs (aspirin), proton pump inhibitors, and statins, are associated with a better response and a longer TTF (time to treatment failure) in patients treated with ICIs (11, 32, 86). There is a lack of incontrovertible evidence about the efficacy of these agents and further studies are essential to investigate their role. Translating these observations to the clinic will require many preclinical and clinical experiments to find optimal synergy with immunotherapeutic treatments of metformin.

The biggest challenges of current ICI treatment are to improve the efficacy of existing immunotherapies and to elucidate the mechanisms of antitumor immune responses in patients with cancer. Metformin has been widely shown to reduce the incidence of various types of cancer and to have positive effects on antitumor treatment. Personalized drug therapy strategies, which aim to provide tailored treatment for individual patients, should be conducted in future clinical trials to identify the patients who will benefit the most from metformin administration. Time will decide whether metformin can become a new “bullet” for patients undergoing ICI therapy. Moreover, the safety, efficacy, and dosage of metformin plus ICIs in cancer treatment still require greater clarification.

## Author Contributions

ZL, WL, and YW wrote this review article. JL and ML did administrative, technical, or material support. WL and ZL designed the study and contribute to the manuscript preparation. All authors contributed to the article and approved the submitted version.

## Funding

This work was supported by the Natural Science Foundation of Hunan Province China (Grant No: 2017JJ3462, 2020JJ5822) and National Natural Scientific Foundation of China (82003883).

## Conflict of Interest

The authors declare that the research was conducted in the absence of any commercial or financial relationships that could be construed as a potential conflict of interest.

## Abbreviations

AHR, aryl hydrocarbon receptor; AMPK, AMP-activated protein kinase; *A. muciniphila*, *Akkermansia muciniphila*; BACH1, haem-binding transcription factor; CD8TIL, CD8+T tumor-infiltrating lymphocyte; CTLA-4, cytotoxic T-lymphocyte antigen-4; DCR, disease control rate; dMMR, DNA mismatch repair destroy; ICIs, immune check inhibitors; irAEs, immune-related adverse effects; MSI-H, microsatellite instability-high; mTOR, mechanistic target of rapamycin; mTORC1, mechanistic target of rapamycin complex 1; NSCLC, non-small cell lung cancer; OR, overall survival; ORR, objective response rate; PFS, progression free survival; PD-L1, programmed death ligand 1; PD-1, programmed cell death receptor-1; SCFAs, short-chain fatty acids; TNBC, triple-negative breast cancer; Tregs, regulatory T cells; T2DM, type 2 diabetes mellitus.
